# Spatial-temporal differences of COVID-19 vaccinations in the U.S.

**DOI:** 10.1007/s44212-022-00019-9

**Published:** 2022-12-19

**Authors:** Qian Huang, Susan L. Cutter

**Affiliations:** Hazards Vulnerability and Resilience Institute, Department of Geography, University of South Carolina, 709 Bull Street, Columbia, SC 29208 USA

**Keywords:** COVID-19, Vaccinations, Urban-rural differences, Spatial nonstationarity, GIS

## Abstract

Although the disparities in COVID-19 outcomes have been proved, they have not been explicitly associated with COVID-19 full vaccinations. This paper examines the spatial and temporal patterns of the county-level COVID-19 case rates, fatality rates, and full vaccination rates in the United States from December 24, 2020 through September 30, 2021. Statistical and geospatial analyses show clear temporal and spatial patterns of the progression of COVID-19 outcomes and vaccinations. In the relationship between two time series, the fatality rates series was positively related to past lags of the case rates series. At the same time, case rates series and fatality rates series were negatively related to past lags of the full vaccination rates series. The lag level varies across urban and rural areas. The results of partial correlation, ordinary least squares (OLS) and Geographically Weighted Regression (GWR) also confirmed that the existing COVID-19 infections and different sets of socioeconomic, healthcare access, health conditions, and environmental characteristics were independently associated with COVID-19 vaccinations over time and space. These results empirically identify the geographic health disparities with COVID-19 vaccinations and outcomes and provide the evidentiary basis for targeting pandemic recovery and public health mitigation actions.

**Supplementary Information:**

The online version contains supplementary material available at 10.1007/s44212-022-00019-9.

## Introduction

While COVID-19 has caused significant global impacts and affected communities unequally, nowhere is this more pronounced than in the U.S. (Crews & Taylor, 2022; JHU, 2022). Despite the social distancing measures, stay-at-home orders, and restricted movement, the global vaccination process also accelerated in response to the COVID-19 pandemic. Safety and efficacy results for vaccines were over 90% against symptomatic and severe diseases caused by contagion of COVID-19 (Anderson et al., 2020; Polack et al., 2020; Voysey et al., 2021). McLaughlin et al. (2022) found that U.S. counties with higher proportions of persons 12 years of age and older and fully vaccinated had lower rates of COVID-19 cases and deaths. According to Moghadas et al. (2021), vaccinations significantly reduced adverse outcomes including ICU hospitalizations, and deaths by 65.6%, and 69.3%, respectively. However, during the Omicron wave, areas with high vaccination rates (over 60%) experienced 1.4 times higher COVID-19 incidence rate but 0.62 times lower mortality rate compared to areas with low vaccination rates (< 40%) (Cuadros et al., 2022). A study simulated the pandemic trajectory under a no-vaccination scenario and suggested that the actual vaccination campaign saved an estimated 240,797 lives and prevented an estimated 1,133,617 hospitalizations from Dec 12, 2020 to Jun 30, 2021 (Vilches et al., 2022).

Spatial and temporal nonstationarity exist when assessing the associations between socio-demographic features and COVID-19 transmission. Specifically, socio-demographic characteristics significantly affecting COVID-19 outcomes or vaccinations in one location or at a time may not be as significant in another location or time period. For example, people’s behaviors change over time in response to changes in their risk perceptions and government mandates such as stay-at-home orders and travel restrictions (Xiong et al., 2020; Kwan, 2021). In mid-2020, state and local governments issued strict stay-at-home orders, consequently human movement decreased significantly during this time. But when COVID-19 cases decreased in spring 2021, states lifted virus restrictions and people relaxed their gathering behaviors, and this relaxed behavior persisted through subsequent waves of COVID-19 (Leatherby, 2021). Another study in Hong Kong also observed how government control measures influenced the spatial distribution of COVID-19 case clusters differently over time (Kan et al., 2021).

Research found a spatial association between vaccination rates and the surge of COVID-19 infections in the U.S. (Cuadros et al., 2022). However, such studies usually aggregate the vaccination rates into levels (e.g., (< 30% and > 50%) in monthly intervals and failed to explain the reason behind it. Moreover, spatial and temporal nonstationarity was not well emphasized for contextual variables related to COVID-19 full vaccination rates. This paper examines how COVID-19 vaccinations align with COVID-19 cases, deaths, and other contextual variables across urban and rural counties in the U.S. from December 24, 2020 through September 30, 2021. This time frame covers the period from the first recorded vaccination cases in the U.S. until just before the Omicron variant appeared. Two questions guide this analysis:

1. What are the time series relationships between COVID-19 daily new cases, fatalities, and vaccinations in the U.S., and how do the patterns vary based on urban-rural location?
2. How do the full vaccination rates relate to case rates, fatality rates, social-demographic characteristics, comorbidities, environmental factors, and healthcare access over space and time?

## COVID-19 disparities and drivers

### Urban-rural disparities of COVID-19

Disparities in COVID-19 outcomes and vaccination coverage between urban and rural locations exist (Centers for Disease Control and Prevention (CDC), 2021a, b; Huang et al., 2021; Murthy et al., 2021). Jackson et al. (2021) suggested that the COVID-19 pandemic initially adversely affected the urbanized Northeast region of the United States, yet as the pandemic progressed the fatality rate in rural areas was higher than in the urban areas. The COVID-19 incidence rate in urban counties was higher between April 1–August 29, 2020, but lower between August 30–November 12, 2020, compared to rural counties (Cuadros et al., 2021). The spatial correlations of new active cases in the U.S. between urban centers were much more significant than between rural areas, and rural populations often have a higher proportion of residents who are over age 65, lack health insurance, live with comorbidities or disabilities, and have limited access to health care facilities as well as factors increasing the risk for COVID-19-related morbidity and mortality (CDC, 2021a; Dobis & McGranahan, 2021; McMahon et al., 2022). Moreover, the predicted case fatality rate (CFR) increased as counties became more rural (Pro et al., 2020). Iyanda et al. (2022) confirmed the statement and suggested that CFR was significantly higher in rural counties with a higher share of Black, indigenous, and people of color (BIPOC) populations in the Northeast and Midwest regions, while political inclination predicted COVID-19 CFR in rural areas in the Midwest region.

Vaccination disparities also exist across the urban-rural continuum (Murthy et al., 2021). From December 14, 2020 through April 10, 2021, vaccination coverage was higher in urban counties (45.7%) than in rural counties (38.9%), and varied among different age groups (adults age 18–64, 65 and older) and gender identities (Murthy et al., 2021). A positive and stronger relationship between area deprivation index (ADI), indicating poor health outcomes and low socioeconomic status, and COVID-19 prevalence was found in rural counties compared with urban ones (Kitchen et al., 2021).

### Drivers of COVID-19 vaccination disparities

Existing inequalities based on social-demographic characteristics such as age, race, median household income, population density, religious affiliation, and political party affiliation contribute to a greater liability of COVID-19 exposure and vaccination, thereby exacerbating health disparities (McPhearson et al., 2020; Raifman & Raifman, 2020; Lin et al., 2022). Age is a determining demographic factor. For example, children had milder clinical symptoms and fewer laboratory and radiologic abnormalities, while older adults had higher mortality rates than any other age groups early in the pandemic (Rozenfeld et al., 2020; Zimmermann & Curtis, 2020). As of August 2022, the rate of death was 140 times higher in the 75–84 age group, and 340 times higher in those 85 years and older when compared to adults 19–29 years old (CDC, 2022a). From December 14, 2020 through April 10, 2021, vaccination coverage was higher in adults age 65 and over than in younger groups (Murthy et al., 2021).

Reitsma et al. (2021) quantify how differential vaccine uptake by race and ethnicity within each U.S. state produced substantial vaccination coverage disparities during the initial scale-up among older adults. In terms of median household income, Sargent et al. (2022) found those with annual household incomes greater than $50,001 were 1.5 times more likely to be vaccinated than those with lower incomes. A survey from Viswanath et al. (2021) also showed that those with lower incomes were less likely to vaccinate people in their care than those with higher incomes.

Furthermore, studies show religious affiliation influences COVID-19 exposure and prevention. On the one hand, many religions require the faithful to congregate which violates the social distancing mitigations (Tan et al., 2022). Religious gathering often involves travel and crowding people at a specific time and location, which may have led to the spread of respiratory disease due to close contact among attendees (Abubakar et al., 2012). On the other hand, as an indicator of social capital, religious affiliation was confirmed to have positive outcomes in many dimensions, such as increasing personal resilience, ability to cope with uncertainty, perceptions of community solidarity, and reported compliance with public health measures (Carter & Cordero, 2022).

Political party affiliation plays an important role in COVID-19 exposure and vaccination. The gap in COVID-19 deaths grew fastest during October 2021 when the death rate in Republican-leaning counties (25 per 100,000 population) was more than three times higher than the rate in Democratic-leaning counties (7.8 per 100,000) (Leonhardt, 2021). Democratic counties consistently had higher vaccination rates than Republican counties with the gap widening steadily by month (Ye, 2021).

Comorbidities and health behaviors such as drinking are also associated with COVID-19 infections and risk perception (Fang et al., 2020). Obesity, diabetes with complications, and anxiety had the strongest association with COVID-19 death. What is more, alcohol consumption is one of the risk factors for the spread of COVID-19 infection by its demonstrated role in reducing social distancing (Gurrieri et al., 2021). Research suggested that increased consumption of alcohol was associated with increases in new COVID-19 infections 2–4 weeks later in Canada (Stockwell et al., 2022).

In addition, both natural and built environments can affect COVID-19 infections and vaccination. The natural amenities such as open space and access to parks are measures of the physical characteristics of a county area that enhance the location as a place to live. Green spaces have become one of the only sources of resilience amidst the COVID pandemic because of their positive effects on physical, psychological, and spiritual wellness (Geng et al., 2021). Access to health care and providers determines the counties’ capacity for sufficient healthcare services. The “availability” of healthcare access/providers illustrates the extent to which facilities have the resources (personnel, number of beds, etc.) to meet the patient’s needs for testing and receiving vaccination (Penchansky & Thomas, 1981).

## Research design and methodology

### Data

The unit of this analysis is counties, the primary spatial unit for operational levels of emergency management and public health data availability. This study includes 3142 counties or county-equivalent places in all 50 states and the District of Columbia. Table 1 lists detailed explanations of the input data, spatial caveats, and initial data manipulations.

**Table 1:** Input variables and sources for county-level analyses

Category	Variable	Description	Data Source	Time frame
COVID-19 outcomes and vaccination	*COVID-19 confirmed cases*	Daily county-level cumulative totals of positive cases	USAFacts (2021)	24 Dec 2020–30 Sep 2021
	*COVID-19 death*	Daily county-level cumulative totals of death	USAFacts (2021)	24 Dec 2020–30 Sep 2021
	*Vaccinated population*	Daily county-level total number of people who are fully vaccinated (have second dose of a two-dose vaccine or one dose of a single-dose vaccine)	CDC-NPCR (CDC, 2021b) & Texas Department of State Health Service (2022)	24 Dec 2020–30 Sep 2021
Contextual variables	*Percentage of people aged 65 and over*	Percentage of population aged 65 years and over	U.S. Census Bureau (2022), ACS 5-Year	2016–2020
	*Population density*	Number of people per square mile	U.S. Census Bureau (2022), ACS 5-Year	2016–2020
	*Median household income*	Median household income (dollars)	U.S. Census Bureau (2022), ACS 5-Year	2016–2020
	*Non-Whites*	Percentage of population not classified as white	U.S. Census Bureau (2022), ACS 5-Year	2016–2020
	*Percentage of voting Democratic*	Percentage of voting Democratic Party for the 2020 U.S. presidential election by counties.	MIT Election Data and Science (Harvard Dataverse, 2021)	2020
	*Religious affiliation*	County-level congregation membership per 100,000 population	US Religious Census (2010)	2010
	*Diabetes*	Age-adjusted prevalence of Diabetes among adults aged > = 18 years	CDC PLACES (2022)	2018–2021
	*Liquor store density*	Number of liquor stores per 100,000 population. A liquor store is defined as a business that primarily sells packaged alcoholic beverages, such as beer, wine, and spirits.	CDC Economic Survey (2020)	2020
	*National amenity scale*	Index for livability of area based on climate factors	USDA-ERS (2019)	2019
	*Environmental hazard*	Total amount of emissions from Toxics Release Inventory (TRIs) in county per square mile	EPA-Toxics Release Inventory (TRI) Program (US EPA, 2013)	2020
	*Primary care provider*	Number of primary care provider per 100,000 population	HRSA Area Health Resources Files (HRSA, 2021)	2020–2021
Location	*Urban-Rural Delineations*	Urban-Rural Classification Scheme for Counties	National Center for Health Statistics (NCHS) (2019)	2013

#### COVID-19 data

Publicly available COVID-19 daily cumulative case and death counts from December 24, 2020 through September 30, 2021 were downloaded from USAFacts on October 1, 2021. USAFacts (2021) data was from the CDC, state- and local-level public health agencies. Cases, deaths, and per capita adjustments reflect cumulative totals since January 22, 2020. All counts for cases (presumptive and confirmed) and deaths (COVID-19 as the primary cause) used patients’ county of residence. For California and Texas, where state public health websites do not offer accurate county-level statistics, USAFacts (2021) individually pulls the most current numbers from each county’s public health website (58 counties for California and 254 counties for Texas). Some cases and deaths counted at the state level were not allocated to counties due to a lack of information, therefore this data (0.28% of the total) was eliminated in the analyses.

COVID-19 daily cumulative vaccinations data from Table 1 was downloaded from the CDC in October 2021. Data on Texas was requested and received from the Texas Department of State Health Service on April 1, 2022. The data includes all vaccine partners including jurisdictional partner clinics, retail pharmacies, dialysis centers, long-term care facilities, Federal Emergency Management and Health Resources and Service Administration partner sites, and federal entity facilities (CDC, 2021b). To better understand COVID-19 vaccination disparities, the county-level vaccine administration data was collected for the population 18 years old and over who were fully vaccinated (completed the primary series of vaccination: had a second dose of the Pfizer-BioNTech or Moderna COVID-19 vaccine or one dose of Janssen COVID-19 vaccine from Johnson & Johnson) during the study period in 51 U.S. jurisdictions (50 states and D.C.). All counts based on the jurisdiction and county were allocated where the vaccine recipient resides. Missing vaccination data on CDC websites was filled out with each state’s public health website data.

#### Contextual variables

The American Community Survey (ACS) 5-year data estimates (2016–2020) were downloaded in August 2022. These estimates obtained socio-demographic variables including total population, percentage of population age 65 and over, population density, median household income, and percentage of the population not classified as White. Election data (percentage of people voting Democratic) was collected from Harvard Dataverse (2021) on October 2021, which is maintained by the MIT Election Data and Science Lab (MEDSL). Religious affiliation measures county-level congregation membership per 100,000 population, collected from US Religious Census (2010). Comorbidity (age-adjusted prevalence of diabetes) and healthcare provider (primary care provider per 100,000 population) were from CDC PLACES (2022) and HRSA Area Health Resources File (2021). Natural and built environmental variables include liquor store density, national amenity scale, and environmental hazard.

#### Urban-rural delineations

All data were matched by county of residence to urban-rural categories according to the 2013 National Center for Health Statistics (NCHS) urban-rural classification scheme (NCHS, 2019). A binary urban/metro (1) (NCHS classes 1–4) and rural/nonmetro (0) (NCHS classes 5–6) for rural for each U.S. county were used in this study.

### Disparity assessment

To set up the background, the 7-day moving averages of COVID-19 daily new case rates, fatality rates, and full vaccination rates were calculated and grouped into three equal length time periods: Period 1: 12/24/2020–3/27/2021 (94 days); Period 2: 3/28/2021–6/29/2021 (94 days); and Period 3: 6/30/2021–9/30/2021 (93 days). For spatial disparities, the case rates (cumulative cases per 100,000 population), death rates (deaths per 100, 000 population), and full vaccination rates (fully vaccinated per 100,000 population) were exhibited for same time periods. The Global and Optimized Local Moran’s I in ArcGIS Pro 2.8.0 (ESRI, Redlands, CA, USA) was utilized to determine the spatial clusters of COVID-19 cumulative case rate, death rate, and full vaccination rate as a measure of spatial autocorrelation. Global Moran’s I provided a single value for the entire nation, and the Local Moran’s I identified spatial clusters and outliers of COVID-19 outcomes. Spatial relationships were inverse distance, and the distance method was Euclidean in Moran’s I analysis.

### Case, fatality, and full vaccination rates time series

This study used a time series analysis method to analyze the cross-correlation between 1) case rates and fatality rates, 2) full vaccination rates and case rates, and 3) full vaccination rates and fatality rates among urban and rural areas. The cross-correlation function (CCF) identifies lags or leads of the two time-series. The CCF is defined as the set of correlations between x_t + h_ and y_t_ for h = 0, ±1, ± 2, and so on (h for lag level). The maximum negative CCF lag means X leads Y; positive lag means X lags Y; and 0 lag means the changes of one variable occur immediately following changes in another variable. In this study, the lag was set to 1-day length, considering that the duration between the date of infection, the onset of severe outcomes, and the effect of full vaccination is usually several days (Colburn, 2021; Puckey, 2022).

### Underlying reasons explanation

To explain the underlying reasons of different distribution of full vaccination rates over time, partial correlations were conducted between full vaccination rates and case rates, fatality rates, and the contextual variables. The contextual variables included percentage of population age 65 and over, diabetes, religious affiliation, liquor store density, natural amenity scale, environmental hazard, primary care provider density, population density, median household income, Democratic Voter, and race (Non-White). Employing RStudio version 2022.07.0, the statistical analysis differentiated urban and rural areas in three time periods as defined previously.

To identify the spatial nonstationarity of these explanatory variables across the U.S. counties, a multivariate regression (ordinary least squares [OLS]) was performed based on 11 independent variables, initially. The model residuals were submitted to spatial dependence analysis by global Moran’s I statistics to assess the need to incorporate a Geographically Weighted Regression (GWR) model. GWR generates a local model for each county, considering the observation values of its neighboring counties. These regression analyses were done with ArcGIS Pro 2.8.0.

## Results

Around 24 million confirmed COVID-19 cases, 346,000 fatalities, and over 170 million full vaccinations occurred in the U.S. during the period of December 24, 2020, through September 30, 2021. The results, organized by the research questions, begin with the spatial and temporal patterns and clusters of cases, fatalities, and vaccinations in three time periods, followed by cross-correlation of daily new case, death, and vaccination rates among urban/rural areas, and ends with the significance of explanatory predictors.

### Setting the background

**Fig. 1:**
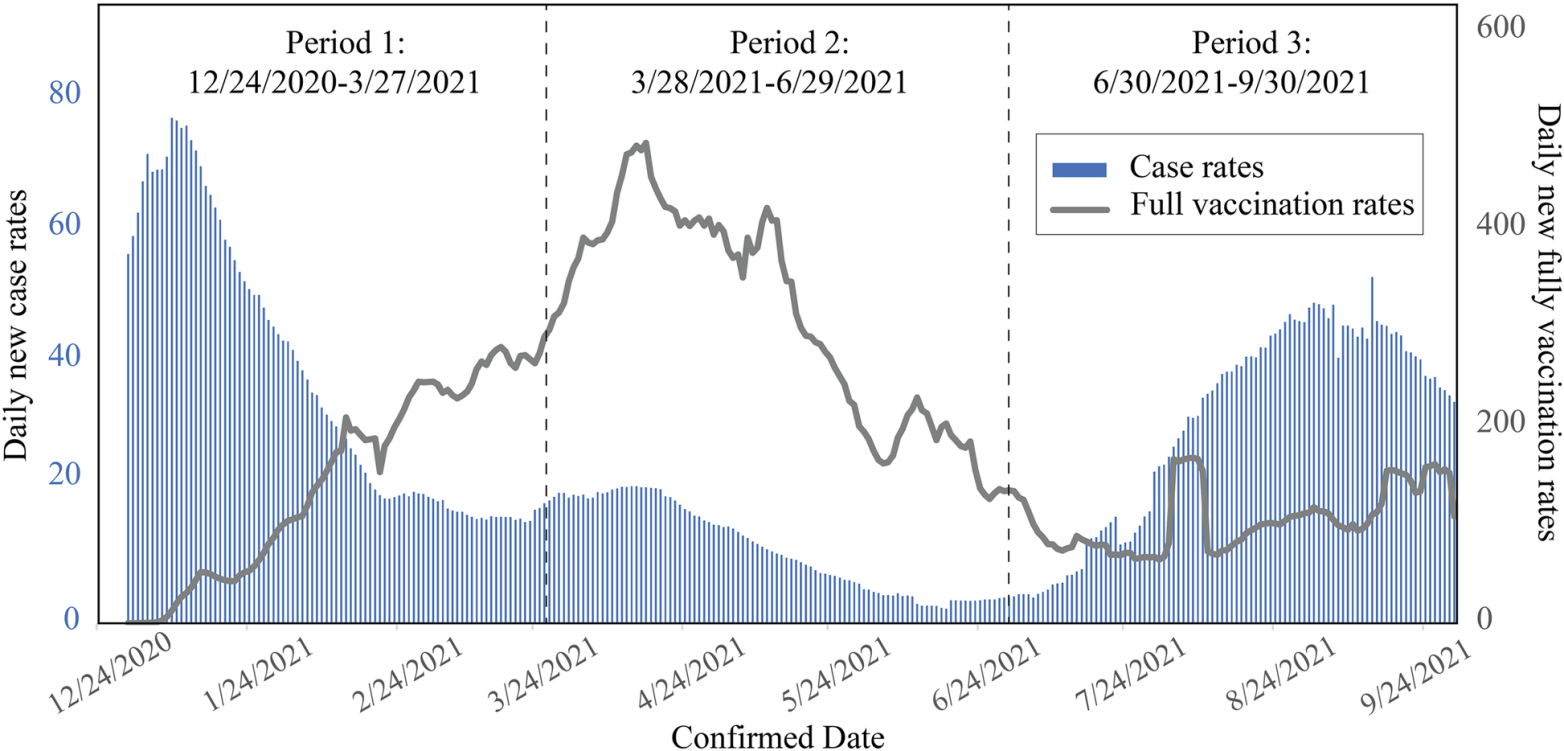
The 7-day moving average of daily COVID-19 new case rates and full vaccination rates in the U.S. from 24 December 2020 to 30 September 2021. This artwork was created in MS Excel and Adobe Illustrator

**Fig. 2:**
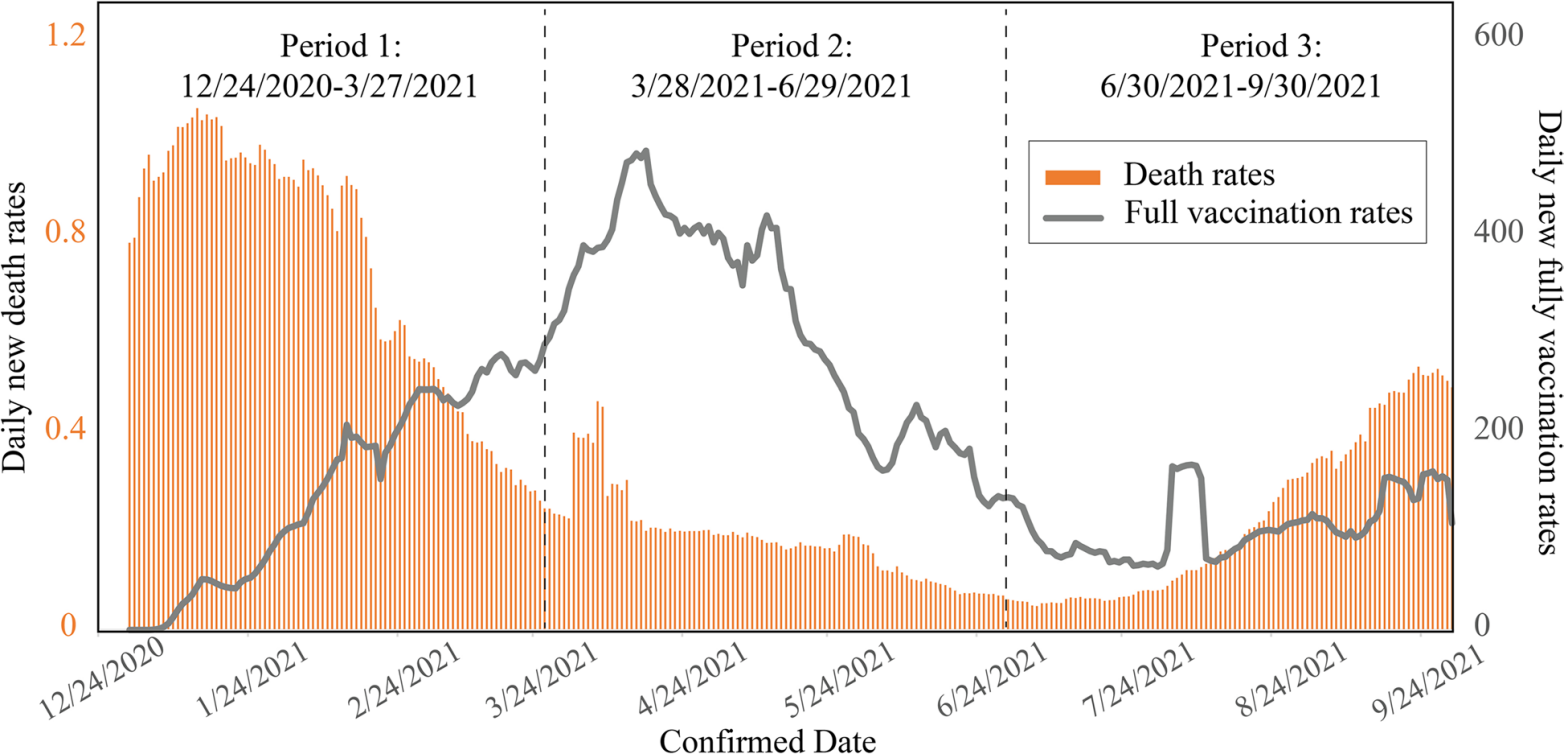
The 7-day moving average of daily COVID-19 death rates and full vaccination rates in the U.S. from 24 December 2020 to 30 September 2021. This artwork was created in MS Excel and Adobe Illustrator

The national trend in 7-day moving average of daily new case rates, death rates, and full vaccination rates, for the study period, exhibited an expected periodicity (see Figs. 1 and 2). The flow of daily new case rates and death rates was more evident in the surge of Period 1 (12/24/2020–3/27/2021), when the initial virus mutated with the new Alpha and Beta variants and spread. Death rates had several days’ delay and soared in Period 1. At the beginning of 2021, the implementation of vaccination programs varies in each state depending on the ability of clinics and availability of vaccine supply (Moghadas et al., 2021). Shortly thereafter, daily case rates and death rates fell as quickly as they had risen in one community after another community in March. A mild surge of new cases happened in Period 2 (3/28/2021–6/29/2021), and vaccination had an enormous surge in winter and spring 2021 when all adults first became eligible for the vaccine. In June 2021, cases and deaths were at their lowest point since the beginning of the pandemic, and a large amount of the population had been fully vaccinated. Because of this decrease, states lifted virus restrictions and people relaxed their behaviors when gathering. A more contagious variant, Delta, quickly grew to account for most U.S. cases and deaths, causing a third wave during Period 3 (6/30/2021–9/30/2021).

**Fig. 3:**
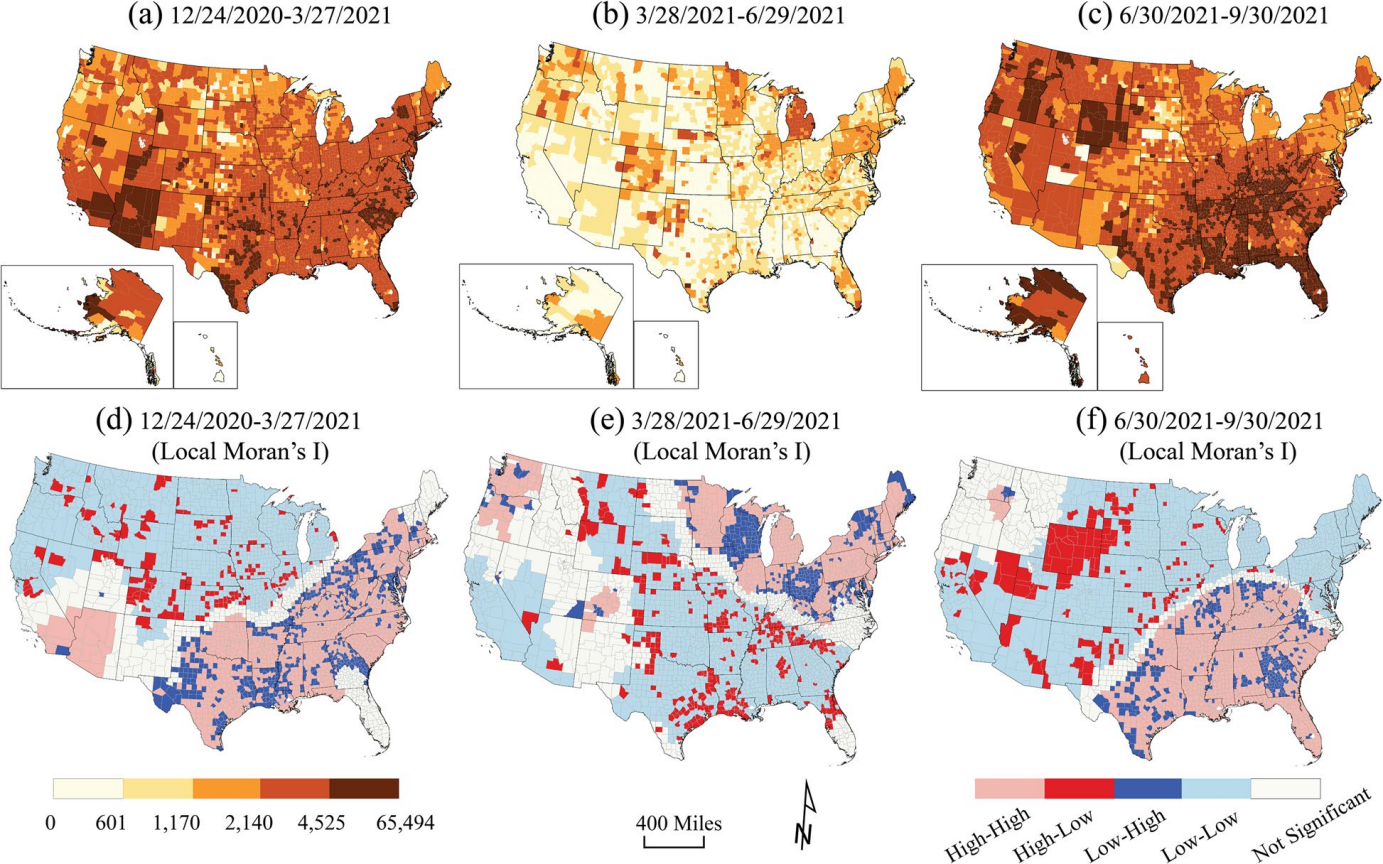
County-level case rates (cases per 100,000 population) during **a** Period 1: 12/24/2020–3/27/2021, **b** Period 2: 3/28/2021–6/29/2021, and **c** Period 3: 6/30/201–9/30/2021, using USCB State and County Boundaries. Local Moran’s I result of case rates during **d** Period 1, **e** Period 2, and **f** Period 3, mapped by high-high cluster (pink), high-low outlier (red), low-high outlier (blue), low-low cluster (light blue), and not significant (white). The artwork was created by ArcGIS Pro 2.8.0 and Adobe Illustrator 2022

Geographically, cumulative case rates from December 24, 2020 through September 30, 2021 showed a varied pattern over time (Fig. 3). The case rates in the first and third periods were significantly higher than in the second period. The clusters of high case rates moved from the South, Southwest, and Northeast in Period 1 to the Midwest and Northeast (Period 2) and then back to the South (Period 3) (Fig. 3). Lower case rates clustered in the North in early 2021, moved to Great Plains states, southeast, and Minnesota (low-high outliers) in spring, and then shifted to the West, Midwest, and Northeast states in Summer 2021.

**Fig. 4:**
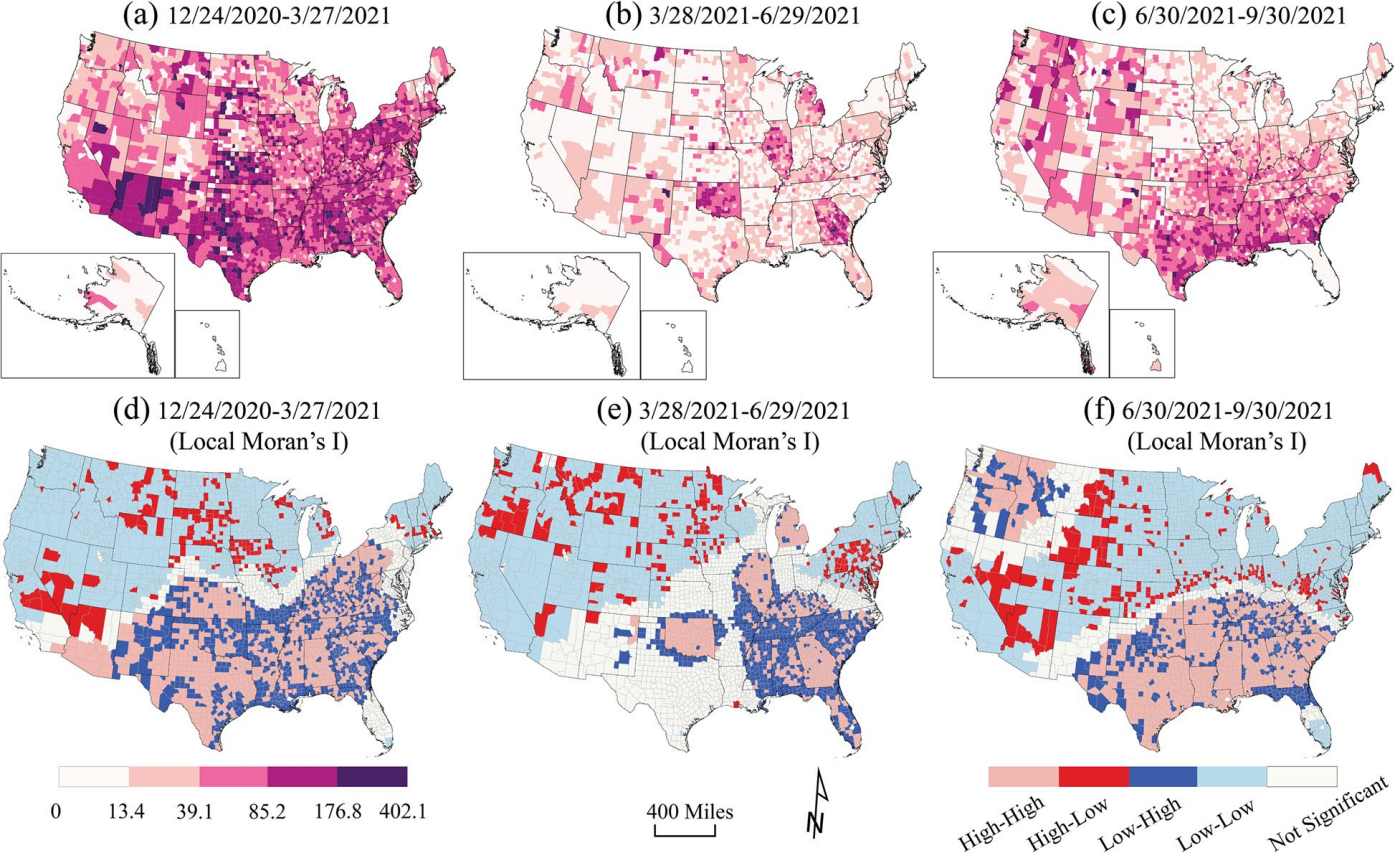
County-level fatality rates (fatalities per 100,000 population) during **a** Period 1: 12/24/2020–3/27/2021, **b** Period 2: 3/28/2021–6/29/2021, and **c** Period 3: 6/30/201–9/30/2021, using USCB State and County Boundaries. Local Moran’s I result of case rates during **d** Period 1, **e** Period 2, and **f** Period 3, mapped by high-high cluster (pink), high-low outlier (red), low-high outlier (blue), low-low cluster (light blue), and not significant (white). The artwork was created by ArcGIS Pro 2.8.0 and Adobe Illustrator 2022

The geographic distribution of high fatality rates (> 176.9 per 100,000 population) were in Kansas, Arizona, Texas, and Alabama during Period 1 (Fig. 4a & d). In period 2, Michigan, Illinois, Arkansas, and Georgia had higher fatality rates than other states. The high-high cluster of fatality rates were in the South and Northwest in Period 3 (Fig. 4c & f). The lowest fatalities (< 13.4 per 100,000 population) were in most of the counties in the West and Northeast in Period 2 (Fig. 4b & e) and the Midwest and Northeast in Period 3.

**Fig. 5:**
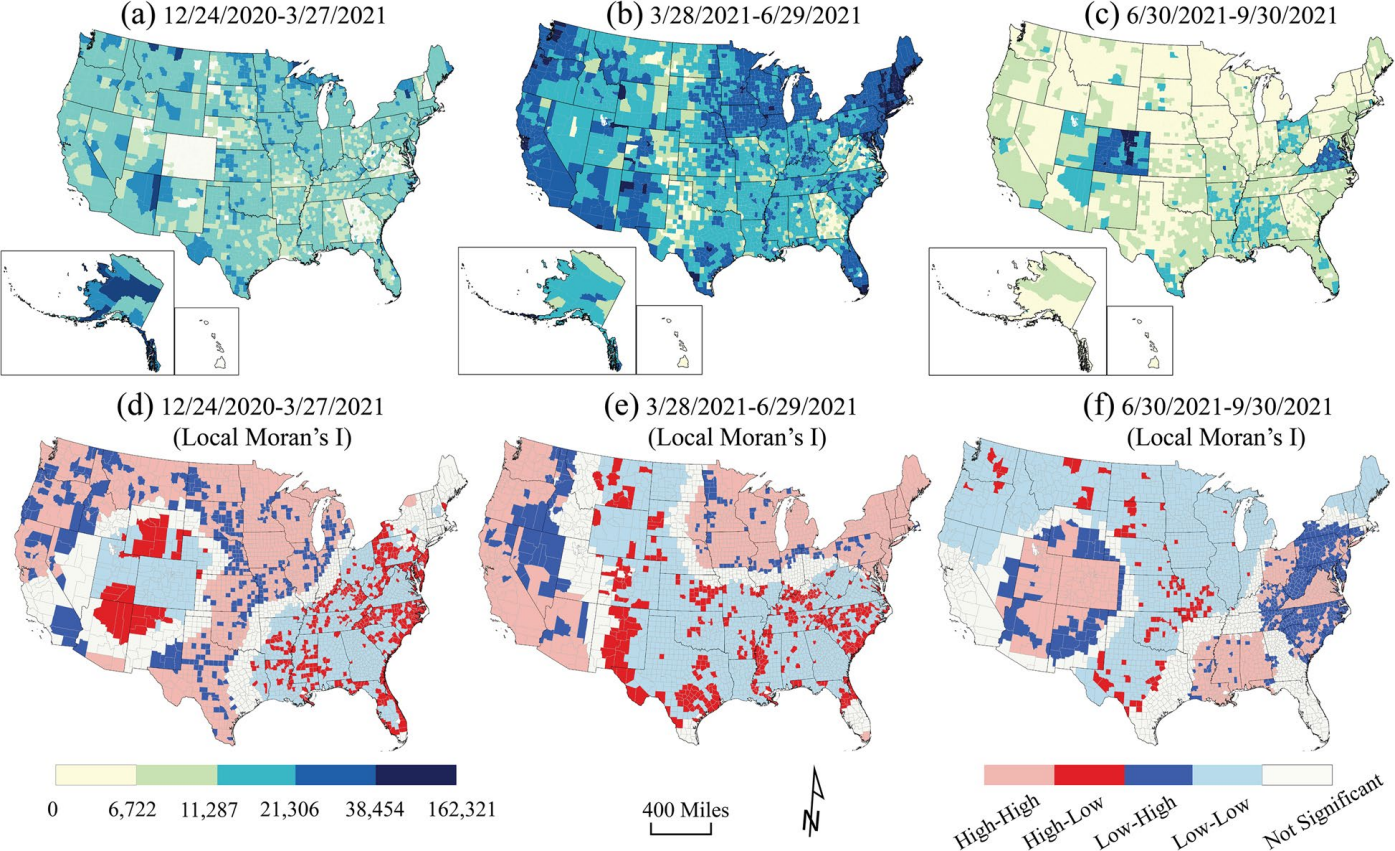
County-level full vaccination rates (fully vaccinated per 100,000 population) during **a** Period 1: 12/24/2020–3/27/2021, **b** Period 2: 3/28/2021–6/29/2021, and **c** Period 3: 6/30/201–9/30/2021, using USCB State and County Boundaries. Local Moran’s I result of case rates during **d** Period 1, **e** Period 2, and **f** Period 3, mapped by high-high cluster (pink), high-low outlier (red), low-high outlier (blue), low-low cluster (light blue), and not significant (white). The artwork was created by ArcGIS Pro 2.8.0 and Adobe Illustrator 2022

Full vaccination rates significantly increased and were highest in the Northwest and the West in Period 2. The lowest full vaccination rates in Period 2 were in the South especially in Georgia and West Virginia. They also occurred in part of the Great Plains states - portions of North Dakota, Montana, Wyoming, and Nebraska, Colorado, Kansas, Oklahoma and Texas (Fig. 5b & e), forming an “L” shape on the map. The patterns in Periods 1 and 3 were reversed: the low-low clustered areas in Period 1 showed a high-high cluster pattern in Period 3, especially in Colorado and Virginia. Positive spatial autocorrelations were observed in the overall case rates, fatality rates, and full vaccination rates for the U.S. (Global Moran’s I = 0.37, 0.31, and 0.36, *p* < 0.0001, respectively).

### Cross-correlation of case rates, death rates, and full vaccination rates

**Fig. 6:**
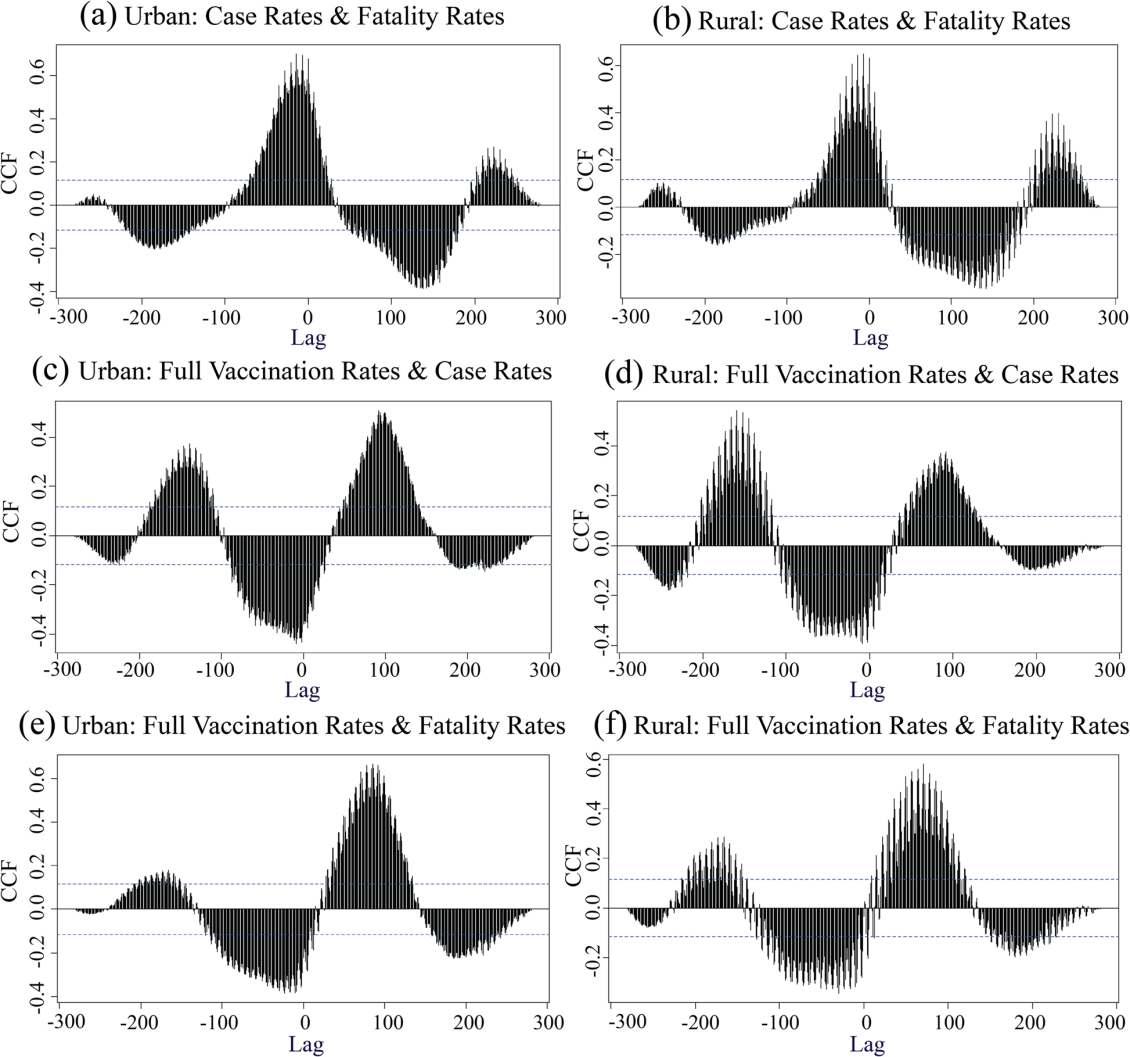
Cross-Correlation Results of **a** Urban case rates and fatality rates, **b** Rural case rates and fatality rates, **c** Urban full vaccination rates and cases rates, **d** Rural full vaccination rates and case rates, **e** Urban full vaccination rates and fatality rates, and **f** Rural full vaccination rates and fatality rates. CCF is cross-correlation function. The artwork was created in R studio and Adobe Illustrator 2022

The first research question asks what are the time series relationships between daily new COVID-19 cases, fatalities, and vaccinations in the U.S., and how these patterns vary based on urban-rural location. Figure 6a & b present the time series cross-correlation of COVID-19 case rates and fatality rates in urban and rural areas. Without considering the time lag (lag level = 0 days), case rates were positively correlated with fatality rates, and this association was strongest in urban areas (cross-correlation coefficient (CCC) in urban = 0.68; CCC in rural = 0.63). The positive correlation was highest when the lag level reaches − 14 days in urban counties (CCC = 0.70, *p* < 0.01) and − 7 days in rural counties (CCC = 0.65, *p* < 0.01). The relationship changed to negative when the lag level was extended to months (urban: < − 94 or > 35; rural: <− 94 or > 23). This means that, statistically, an increase in case rates lead an increase in fatality rates in both urban and rural counties even considering a lag of 4 months, and rural areas had a shorter lag time for the strongest positive correlation (7 days compared to 14 in urban areas).

Figure 6c & d illustrate the cross-correlation of the full vaccination rates and case rates. Results suggest that the full vaccination rates were negatively correlated with the case rates over the lag level of zero days, and this was also strongest in urban areas (CCC_*Urban*_ = − 0.36; CCC_*Rural*_ = − 0.25). The negative correlation was higher at the lag level of − 9 days in both urban and rural counties (CCC_*Urban*_ = − 0.44; CCC_*Rural*_ = − 0.39). This means that increasing full vaccination rates lead a decline in case rates, and this relationship was most pronounced 9 days after receiving full vaccination. In addition, CCC becomes positive as the lag increases to 35 or decreases to − 102 in urban counties, indicating that the high case rates can lead increased vaccination rates on later days.

Similarly, the cross-correlation of full vaccination rates and fatality rates also shows a negative correlation when there was no lag, with a stronger association in the urban counties (CCC_*Urban*_ = − 0.22; CCC_*Rural*_ = − 0.03). The negative correlation was the strongest at the lag level of − 23 days in urban areas (CCC_*Urban*_ = − 0.38) and − 30 days in rural areas (CCC_*Rural*_ = − 0.34). Full vaccination rates lead to decreased fatality rates and this relationship was most pronounced 23 days (urban) or 30 days (rural) after receiving full vaccination. On later days, the cross-correlation shifts to positive, and the strongest positive level was when the lag increases to 85 in urban areas (CCC = 0.66, *p* < 0.01) and 71 in rural areas (CCC = 0.58, *p* < 0.01).

To summarize, case rates and death rates positively correlate in both urban and rural contexts with a short time lag as expected. Increased full vaccination rates lead a decrease in case rates and death rates with moderate strength of correlation. The lag level varies across urban and rural areas, with stronger statistical relationships in urban areas than in rural areas.

### Role of explanatory variables in COVID-19 full vaccination rates

To answer the second research question, two approaches were used to examine the influence of context on the temporal and spatial distribution of full vaccination rates. Findings from each of these are below.

#### Temporal nonstationarity effects

To explore the driving factors of the distinct spatial patterns of full vaccination rates among counties in three periods, partial correlations were conducted between full vaccination rates, case rates, fatality rates as well as other contextual variables controlling for urban and rural areas. Some variables had consistent effects. For instance, case rates had positive impacts on full vaccination rates over time in urban counties (Table 2). Political affiliation was positively and significantly correlated with vaccination rates over space and time, indicating that counties with more Democratic voters had higher full vaccination rates.

**Table 2:** Partial Correlation Results between full vaccination rates and explanatory variables among urban and rural counties in the U.S.

Variable	Urban (***n*** = 1166)			Rural (***n*** = 1976)		
	Period 1	Period 2	Period 3	Period 1	Period 2	Period 3
**Case rates (Periods 1/2/3)**	0.121***	0.302***	0.083**	0.086***	0.065**	0.044
**Fatality rates (Periods 1/2/3)**	− 0.076*	− 0.115***	− 0.063*	0.029	− 0.053*	0.009
**Age over 65**	0.181***	0.002	−0.037	0.117***	− 0.081***	− 0.109***
**Diabetes**	−0.025	0.050	0.026	−0.020	−0.106***	− 0.061**
**Religious affiliation**	0.024	−0.068*	0.081**	0.114***	0.017	0.055*
**Liquor store density**	0.013	0.032	−0.069*	− 0.085***	0.043	0.068**
**Natural amenity scale**	−0.068*	0.082**	0.079**	−0.141***	0.009	0.235***
**Environmental hazard**	−0.065*	−0.077**	0.081**	0.006	−0.048*	0.049*
**Primary care provider**	0.170***	0.018	0.008	0.148***	0.065**	−0.026
**Population density**	−0.057	0.003	0.036	−0.221***	−0.179***	0.283***
**Median household income**	−0.032	0.183***	0.095**	0.087***	0.036	−0.094***
**Democratic voter**	0.110***	0.374***	0.072*	0.172***	0.574***	0.133***
**Race - Non-Whites**	−0.105***	− 0.216***	−0.003	0.007	−0.313***	0.069**

Period 1: 12/24/2020–3/27/2021; Period 2: 3/28/2021–6/29/2021; and Period 3: 6/30/201–9/30/2021

***p < 0.001; **p < 0.01; *p < 0.05

Some factors had temporal nonstationarity effects on measuring COVID-19 vaccination rates. For example, in rural areas, the percentage of the population age 65 and over associated positively with vaccination rates in early 2021 but negatively correlated in later study periods. The natural amenity scale was negatively correlated with vaccination rates in Period 1 across the counties, but the correlation changed to positive in Period 3. Moreover, the density of primary care providers was only significant in Period 1 but not later.

Diabetes, liquor store density, population density, median household income, and race showed urban-rural nonstationarity on full vaccination rates. For instance, the age-adjusted prevalence of diabetes was negative and significant in rural areas but not in urban areas. High population density significantly decreased vaccination rates before the end of June but increased vaccination rates during the summer in rural counties, with no significance in urban counties.

#### Spatial nonstationarity effects

**Fig. 7:**
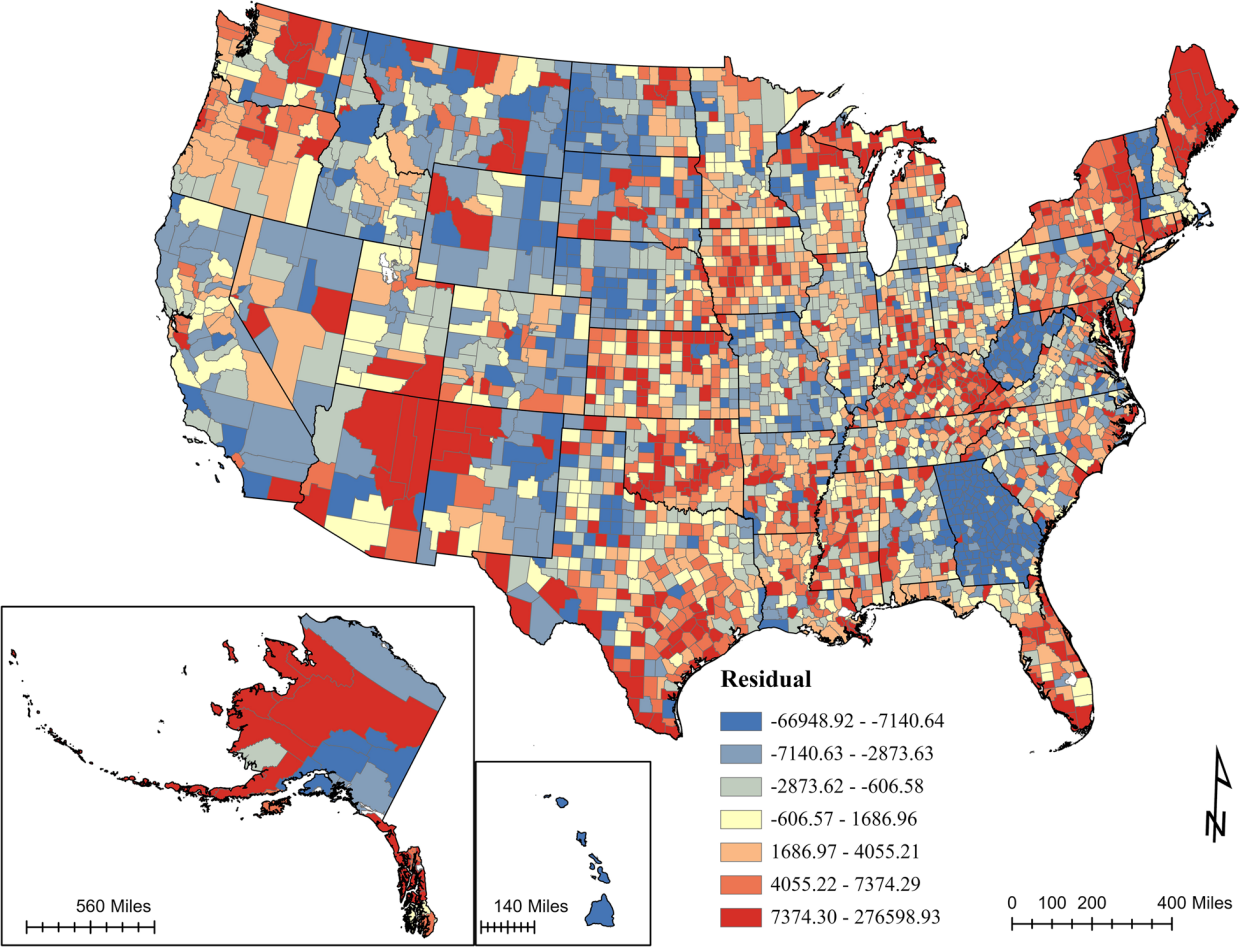
Map of OLS regression residuals. The artwork was created by ArcGIS Pro 2.8.0

An ordinary least squares (OLS) was conducted based on 11 exploratory variables, initially (see details in Supplemental Information 1). Among all significant variables, COVID-19 case rates, religious affiliation, natural amenity scale, primary care provider, median household income, and Democratic voters were positively correlated with full vaccination rates, while COVID-19 fatality rates, environmental hazards, population density, and non-Whites were negatively correlated with full vaccination rates. The model showed moderate prediction power (adjusted R-squared = 0.40, *p* < 0.001) and positive spatial autocorrelation (Moran’s I = 0.12, z = 65.76, *p* < 0.001) (Fig. 7).

**Fig. 8:**
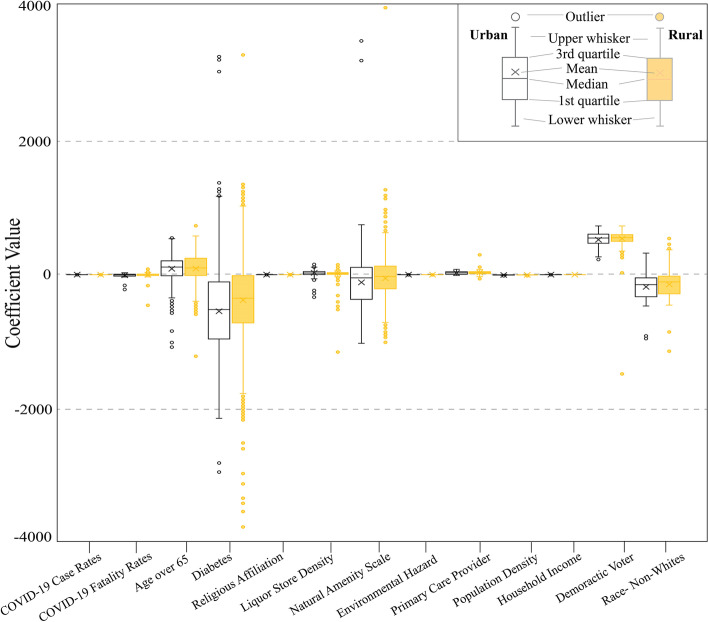
Boxplot of the coefficient values for the 11 independent variables among urban and rural counties. The artwork was created by MS Excel and Adobe Illustrator 2022

To mitigate the issue of spatial autocorrelation, GWR was used to generate a local model for each county. Figure 8 uses box plots to represent ranges of coefficients for 11 independent variables for urban and rural counties. The box in the middle represents 25 percentiles to 75 percentiles, and the bars extend from the minimum to maximum value of each variable. Most of the coefficients range from negative to positive, except for Democratic voters and population density. Democratic Voting was positively related while population density was negatively related to the fully vaccinated population in nearly 100% of counties. There was no significant difference between urban and rural for each variable, but a clear difference between independent variables. For example, the population aged 65 and over was significantly lower than political party affiliation but higher than other variables. Moreover, in urban counties, religious affiliation has the smallest range (from − 0.053 to 0.168) and diabetes has the largest range (from − 2948.080 to 3254.946). In rural counties, median household income has the smallest range (from − 0.101 to 0.342) and diabetes has the largest range (from − 5824.749 to 4339.095). This indicates that the coefficients of diabetes were more dispersed than other variables, and diabetes in urban counties had more scattered data and fewer potential outliers. In addition, most of the box plots are symmetric distributed, while the data sample of natural amenity scale and race were negatively skewed. Such ranges demonstrate that spatial nonstationarity exists among all independent variables across the counties. In other words, the correlation of different exploratory factors and full vaccination rates vary across space, regarding both direction and magnitude.

**Fig. 9:**
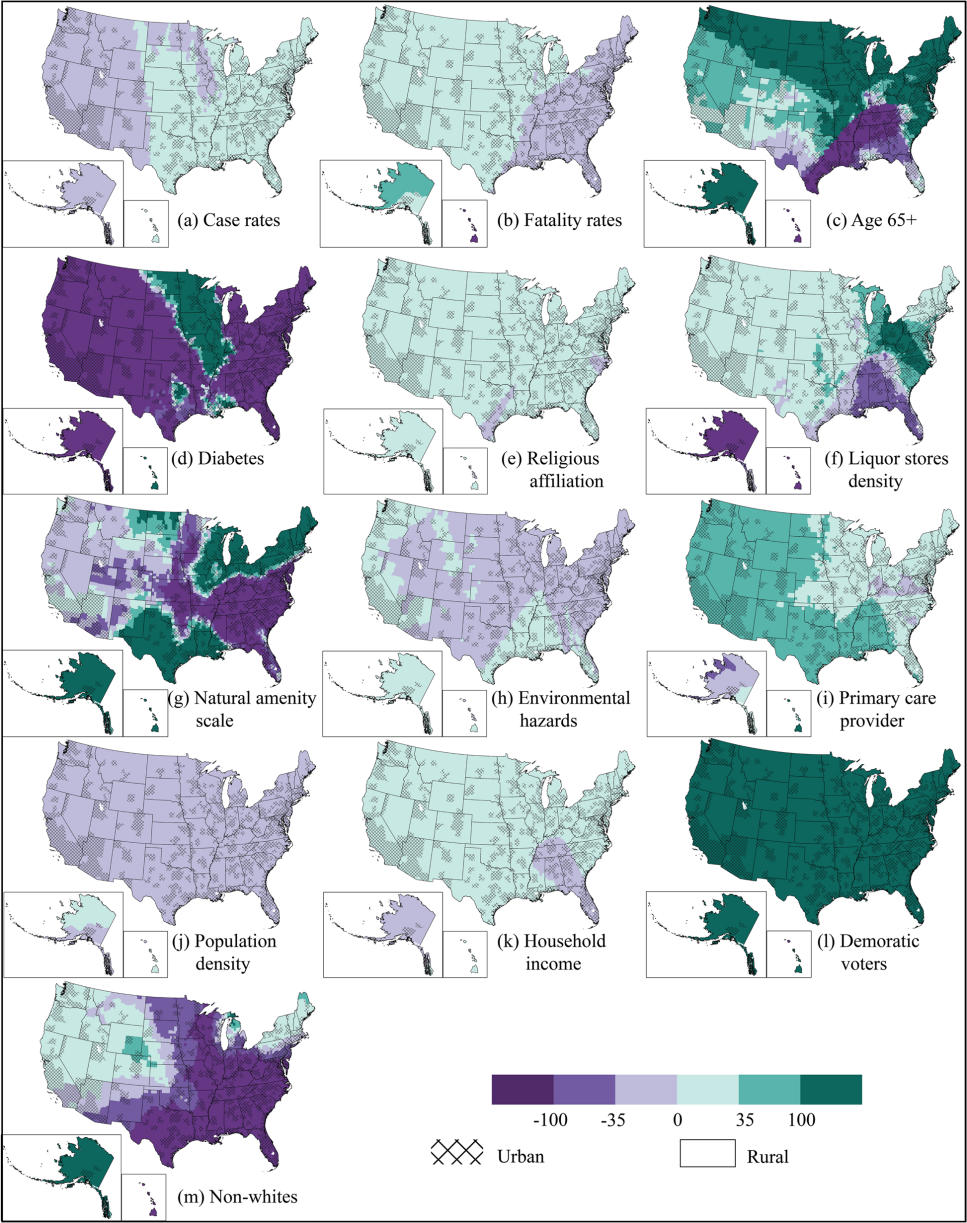
Spatial distribution of the coefficients of the 11 variables. All maps use the same color scheme, so the direction and magnitude of the variables are comparable. The artwork was created by ArcGIS Pro 2.8.0 and Adobe Illustrator 2022

Figure 9 shows local variations of the 11 independent variable coefficients. Patients with diabetes had the lowest full vaccination rates, reflected in Fig. 9d as most of the counties are dark purple (negative correlation). A survey in Saudi Arabia assessed COVID-19 vaccine acceptance and hesitancy rate among patients with diabetes and suggested that participants are willing to vaccinate but show some fear and misinformation (Aldossari et al., 2021). In addition, as Fig. 9m indicated, the non-White population had low full vaccination rates in the South (especially in urban areas), and high vaccination rates in the West. This is consistent with existing literature as well (Reitsma et al., 2021).

Interestingly, case rates were negatively correlated with vaccination rates in the West, but positively correlated in the East (Fig. 9a). Fatality rates had an opposite distribution: positive in the West while negative in the eastern states, with low coefficients (Fig. 9b). The spatial non-stationarity phenomenon is more obvious in the population age 65 and over, diabetes, liquor store density, natural amenity scale, and non-Whites groups (Fig. 9c, d, f, g & m). Religious affiliation was widely positive among counties (Fig. 9e), contradicting to the research that suggested Christianity was negatively related to vaccination rates (Trepanowski & Drążkowski, 2022). Primary care provider density and median household income had widely positive effects (Fig. 9i & k) while environmental hazards and population density had mostly negative effects (Fig. 9h & j), echoing the existing literature (Jung & Albarracín, 2021; Johnson, 2022; Sanchez & Wilkinson, 2022).

**Fig. 10:**
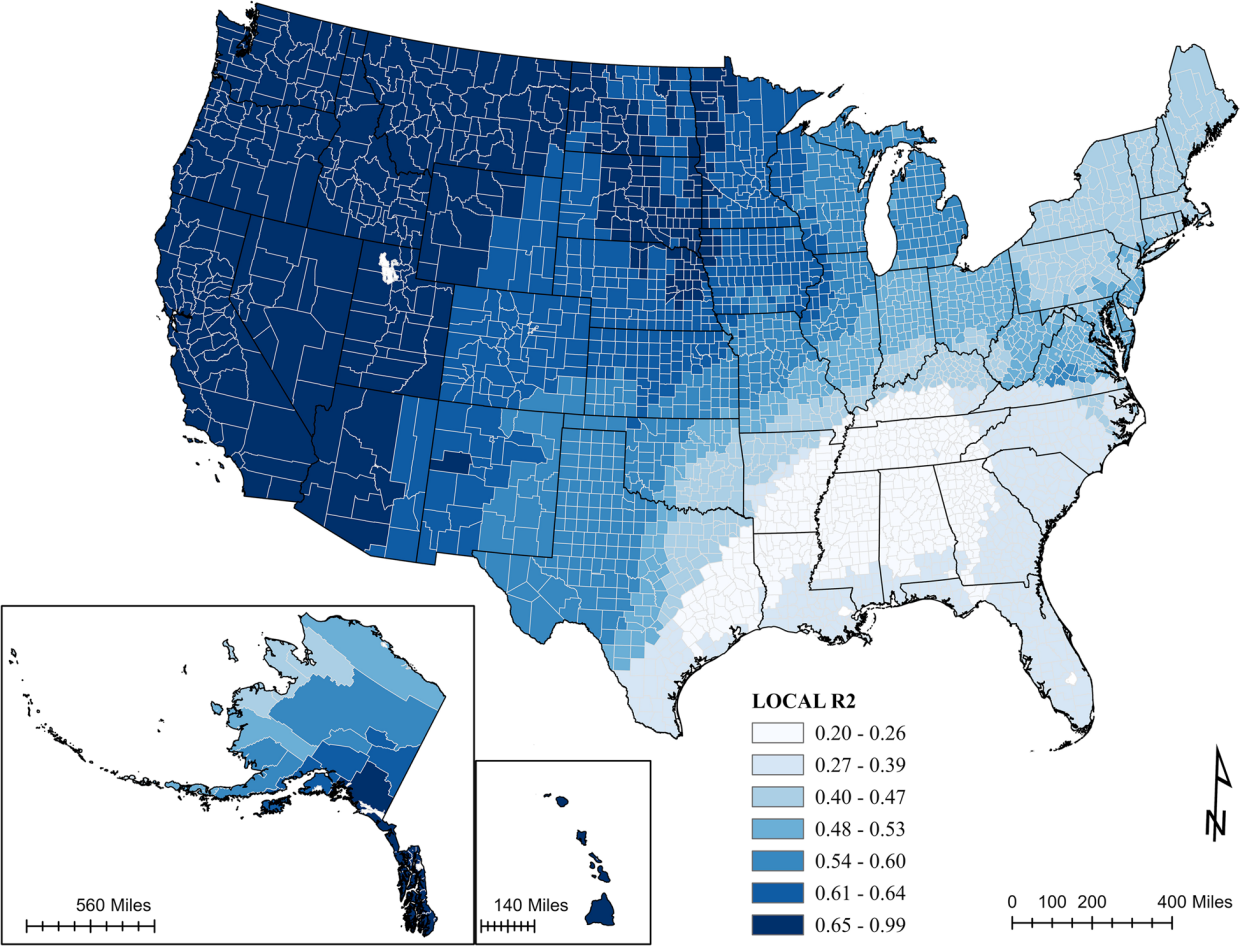
Distribution of R-squared of the GWR results. The artwork was created by ArcGIS Pro 2.8.0

R-squared values of the 3142 local models range from 0.197 to 0.989 with an average of 0.4782 (Fig. 10). In general, R-squared is high in the West and lower in the South. The GWR model has better prediction performance in rural areas than in urban areas.

## Discussion and conclusion

This study extends the evidence of significant differences in the spatial distribution of outcomes and vaccinations in the U.S. from December 2020 to September 2021. There are clear temporal and spatial patterns of the progression of COVID-19 in the U.S. culminating in differential impacts between locations. Concerning overall patterns, the flow of cases and fatalities had a surge in January and mid-February 2021, and the wave of fatalities extended to mid-March. There were independent outbreaks at the same time in disparate communities because of similar school opening dates or similar behavior patterns (Leatherby, 2021). Later, cases and deaths fell as quickly as they had risen in one community after another in March. The sharp fall seen after the winter peak is not an uncommon event, this is common during epidemics after a virus rapidly spreads through a community and runs out of people to infect. A mild surge happened in April. During that period, Michigan saw a large surge in cases and deaths when vaccines were not widely available, non-COVID-19-related patients were being admitted, flu cases were emerging, and health systems were understaffed (Deliso, 2021). Vaccination had an enormous surge in Winter and Spring 2021. In June, cases and deaths were at the lowest point since the beginning of the pandemic, and a high percentage of the population had been fully vaccinated. Consequently, states lifted virus restrictions and people relaxed their gathering behaviors. Another variant, Delta, quickly grew to account for most U.S. cases and deaths, causing a peak in the fall season especially in Missouri and Florida (Leatherby, 2021).

The cumulative COVID-19 case rates, fatality rates, and vaccination rates vary across the U.S. over time and space. The case rates were high in the South and Northeast before April 2021 and moved to the northeast in Spring 2021 and then back to the South in the summer. The high fatality rates had a similar pattern to the case rates since the areas with more cases have a higher risk for severe outcomes. However, Florida showed a clear cold spot for fatalities in Summer. According to reports from the *Miami Herald*, Florida’s methods for reporting COVID-19 information were causing the spread of misinformation and misleading death numbers in early August 2021 (Blaskey et al., 2021). The state switched to reporting deaths by date of death, which was more involved and caused weeks-long delays in reporting. This caused a false “artificial decline” weeks later (Figueroa, 2021). Full vaccination rates were high in the West except in Colorado areas and then increased in the Northeast and western coastal counties in Spring 2021.

In the relationship between two time series, the fatality rates series was positively related to past lags of the case rates series. At the same time, case rates series and fatality rates series were negatively related to past lags of the full vaccination rates series. The lag level varies across urban and rural areas, and correlations were stronger in urban areas. This confirmed the general pattern of COVID-19 outcomes and prevention: high case-high fatality, high vaccination-low case, and high vaccination-low fatality (Huang et al., 2021; Jackson et al., 2021; Cuadros et al., 2022; Suthar et al., 2022).

The study confirmed the temporal nonstationarity effects of contextual variables on COVID-19 full vaccination rates. For example, the percentage of the population age 65 and over and the density of primary care providers were associated positively with vaccination rates in early 2021 but associated negatively in later study periods. This is related to the prioritization of vaccines at early time, which was prioritized for healthcare workers and high-risk individuals, including those with comorbidities related to severe COVID-19, and individuals aged 65 and older (Garnier-Crussard et al., 2020; Wang et al., 2020; Yang et al., 2020).

Similarly, the non-White population had lower full vaccination rates before July 2021 but it increased later. This might involve persisting health disparities among social groups. In general, non-whites are vulnerable as they have less access to healthcare facilities and lower social economic status. Hill and Artiga (2022) suggested that Hispanics represent a larger percentage of cases relative to their percentage of the total population (24% vs. 18%) as of October 2021. The varied effects of the race variable on vaccination rates echo the existing studies. Moyce et al. (2021) conducted 14 semi-structured interviews with 20 Latino participants living in the rural community and suggested that the responders had a very low fear of the virus but a high risk perception of financial loss. Another study conducted by Karout et al. (2020) in minority groups in the U.S. also suggested participants’ low risk perception scores and low engagement in preventive behaviors. But later, with vaccine equity efforts promoted by CDC, and other national, state, tribal, territorial, and community partners, minority groups built confidence in vaccines and raised their awareness of risks (CDC, 2022b). De Bruin and Bennett (2020) conducted an extensive cross-sectional online survey with 6684 adults in the U.S. and suggested that perceiving greater risks were linked with the implementation of protective behaviors. A similar study in Australia showed that people’s risk perception of COVID-19 had a significant impact on whether or not they were practicing health-protective behaviors and vaccination intentions (Faasse & Newby, 2020). Three other studies from Bolivia, China, and Italy also indicated that higher risk awareness was related to greater intention to be vaccinated (Vai et al., 2020; Wang et al., 2020; Zeballos Rivas et al., 2021).

All explanatory variables of full vaccination rates have spatial nonstationarity effects. For example, diabetes, liquor store density, and population density were more significant in rural counties than in urban counties. Religious affiliation decreased the full vaccination rates in southern Texas and eastern North Carolina but increased the vaccination rates in other areas. The positive function of religious affiliation on vaccination rates in this study may be owing to the intention of attending the in-person gathering and religions’ positive impacts on perceptions of community solidarity, and compliance with public health measures (Carter & Cordero, 2022).

Surprisingly, political affiliation positively and significantly correlated with full vaccination rates over time across the counties. During the pandemic’s early months, COVID-19 cases and fatalities were higher in Democrat locations, likely attributed to the several main international transportation hubs such as in New York City and Los Angeles. The virus entered the U.S. from the West and Northeast and swept across the country by the end of 2020 (Leonhardt, 2022). After vaccines became widely available, liberals were much more willing to receive one than conservatives, making COVID-19 a disproportionately Republican illness (Leonhardt, 2022). This partisan gap in COVID-19 deaths has been growing since early 2021. The red states’ perturbations about vaccine side effects have overwhelmed the fears about a deadly virus, which stems from disinformation — promoted by right-wing media, like Fox News and the Sinclair Broadcast Group — that use “distrust” in government and science (Leonhardt, 2021). A recent study concluded that the COVID-19 pandemic has killed more Republicans than Democrats because of Republicans’ opposition to public health measures, particularly mask mandates and vaccination requirements (Risen, 2022).

This study is not without limitations, however. First, some cases and deaths counted at the state level are not allocated to counties due to lack of information, and that data was eliminated in the analysis, possibly affecting the results of the analyses. Second, county units may be too coarse a spatial scale to ascertain linkages between the relationships and the correlates. A subsequent sub-county level such as Zip Code Tabulation Areas (ZCTA) analyses would parse the urban-rural differences more effectively. Third, this study explored temporal nonstationarity by aggregating data into three time periods. Different aggregation, segmentation, and time boundary can be used to further analyze the nonstationarity effects. For example, future studies could use a finer spatial-temporal analysis such as SaTScan to show the cluster full vaccination rates at different time periods.

There are some unique strengths of this study. First, this study extended the evidence of urban-rural disparities in the time series relationship between COVID-19 outcomes and vaccinations in the U.S. Second, this is the first study that has daily vaccination data from Texas between December 24, 2020 through September 30, 2021. In addition, unlike other analyses, this paper employed OLS and GWR to uncover hidden COVID-19 full vaccination patterns in 281 days at the county-level. It also certified that GWR has better prediction performance than OLS in spatial autocorrelation data. Finally, this study confirmed the spatial and temporal nonstationarity of socioeconomic, healthcare access, existing health conditions, and environmental variables impacts on COVID-19 vaccinations, meaning different variables have varying impacts on compliance with public health measures over space and time (Kitchen et al., 2021). This work could raise awareness about health disparities, engage others in conversations about the problems and solutions, guide efforts to promote programs and policies for change, and help individuals and organizations address health issues in their communities. Disparities in COVID-19 outcomes and vaccination between urban and rural communities can hinder progress toward ending the pandemic. Public health practitioners should continue to work with healthcare providers, pharmacies, community-based organizations, faith leaders, and local workers to address vaccine hesitancy and ensure equitable access and distribution of vaccination boosters, especially in rural counties and minority groups (Murthy et al., 2021). The collaborative efforts can not only help improve nationwide vaccination coverage but also reduce benefit resource allocating, thereby aiding health improvement by alleviating COVID-19 disparities in both the short and long terms. Future research directions based on the results of this study could explore the COVID-19 outcomes and vaccination patterns after September 2021 and look deeper at the dynamics of COVID-19 spatial diffusion at different age groups considering the social, behavioral, environmental, health care access, and political contexts.

## Supplementary Information

**Additional file 1.** Summary of OLS Results – Model Variables.

## Data Availability

The datasets generated during and/or analyzed during the current study are available from the corresponding author upon reasonable request.

## Abbreviations

ACS: American Community Survey
ADI: Area Deprivation
BIPOC: Black, Indigenous, and People of Color
CCC: Cross-Correlation Coefficient
CDC: Centers for Disease Control and Prevention
CFR: Case Fatality Rate
GWR: Geographically Weighted Regression
MEDSL: MIT Election Data and Science Lab
NCHS: National Center for Health Statistics
OLS: Ordinary Least Squares
ZCTA: Zip Code Tabulation Areas
