# Supplementary material for: Spatial-temporal differences of COVID-19 vaccinations in the U.S
Source: Urban Inform. 2022 Dec 19;1(1):19. doi: 10.1007/s44212-022-00019-9 (PMC9760536; doi:10.1007/s44212-022-00019-9)
Supplement: Supplementary file 1 — Additional file 1. Summary of OLS Results – Model Variables. [file 44212_2022_19_MOESM1_ESM.docx]

*Article title: Spatial-temporal differences of COVID-19 vaccinations in the U.S.; Journal name: Urban Informatics; Author names: Qian Huang & Susan L. Cutter; Affiliation: University of South Carolina; E-mail: qh1@email.sc.edu*

Supplemental Information 1. Summary of OLS Results – Model Variables

| Variable | Coefficient | Std Error | t-Statistic | Probability | VIF |
| --- | --- | --- | --- | --- | --- |
| Intercept | 23501.86065 | 2787.442036 | 8.431336 | 0.000000* | -------- |
| COVID-19 case rates | 0.02716 | 0.008628 | 3.147743 | 0.001676* | 1.278953 |
| COVID-19 fatality rates | -6.876465 | 2.759693 | -2.49175 | 0.012751* | 1.460304 |
| Age over 65 | 34.289218 | 46.417084 | 0.73872 | 0.460124 | 1.460591 |
| Diabetes | -308.560439 | 149.339549 | -2.066167 | 0.038883* | 3.538004 |
| Religious affiliation | 0.039788 | 0.010845 | 3.668795 | 0.000261* | 1.137081 |
| Liquor store density | 8.980447 | 17.059908 | 0.526407 | 0.598654 | 1.101511 |
| Natural amenity scale | 230.231494 | 87.392518 | 2.634453 | 0.008463* | 1.143382 |
| Environmental hazard | -0.028416 | 0.016795 | -1.691929 | 0.090769 | 1.012518 |
| Primary care provider | 27.600371 | 5.108391 | 5.402948 | 0.000000* | 1.373825 |
| Population density | -0.292191 | 0.143549 | -2.035487 | 0.041876* | 1.21678 |
| Median household income | 0.095568 | 0.019052 | 5.016185 | 0.000001* | 2.279397 |
| Democratic voter | 503.794389 | 18.510076 | 27.217305 | 0.000000* | 2.540077 |
| Race- non-White | -206.076078 | 19.249687 | -10.705425 | 0.000000* | 3.07653 |
